# Commercial inpatient hospital price growth driven by system affiliation and nonprofit-status hospitals

**DOI:** 10.1093/haschl/qxae140

**Published:** 2024-11-01

**Authors:** Jessica Y Chang, Kathryn Martin

**Affiliations:** Health Care Cost Institute, Washington, DC 20005, United States; Health Care Cost Institute, Washington, DC 20005, United States

**Keywords:** health care prices, inpatient prices, hospitals

## Abstract

As policymakers continue to grapple with rising health care costs and prices, understanding trends and variations in inpatient prices among hospital characteristics is an important benchmark to allow policymakers to craft targeted policies. In this study, we provide descriptive trends on variation in inpatient prices paid by commercial health plans stratified by hospital characteristics using data from Health Care Cost Institute's employer-sponsored insured claims data. Our analyses found evidence of considerable variation among inpatient price levels and growth among system affiliation and profitability. Prices among system-affiliated hospitals grew from $14 281.74 in 2012 to $20 731.95 in 2021, corresponding to a 45.2% increase during this period. On the other hand, prices among independent hospitals grew more slowly, from $13 460.50 in 2012 to $18 196.90 in 2021, corresponding to a 35.2% increase. We did not observe a similar trend in growth rates among case mix index by hospital characteristics, implying that differential inpatient price growth is not driven by changes in case mix by hospital characteristics. Heterogeneity in hospital prices and price growth by type of hospital suggests that public and private policymakers aiming to rein in health spending should consider policies that address this variation.

## Introduction

Spending on hospital services makes up more than half of per-person health care spending.^1^ In 2021, inpatient facility spending among enrollees with employer-sponsored insurance alone was nearly 20% ($1245 per person) of total per-person health spending and had grown by 19.7% since 2017, an increase that was driven by increasing prices in spite of lower health care utilization rates.^1^ Compared with public payers, prices paid by private payers are considerably higher, and price is one of the factors contributing to year-to-year increases in health care spending growth.^2^ Mergers and consolidations among hospitals also have increased during this same time period. Consolidations increased for all hospital service lines, such as obstetrics and intensive care units.^3,4^ Previous literature has found that mergers and consolidations lead to higher inpatient prices paid by commercial payers.^5,6^

As state and federal policymakers look to lower health care costs, hospitals’ significant role in the health care ecosystem demands their attention. At the same time, not all commercial inpatient hospital prices or price growth are the same. Inpatient hospital prices vary by a number of factors, including geography, type of service, and hospital characteristics. In this article, we provide a national descriptive analysis of trends in commercial inpatient prices between 2012 and 2021 among hospital characteristics, such as profitability and health system affiliation. We found a wide and growing difference in commercial inpatient prices by type of hospital, particularly between system-affiliated hospitals and their independent hospital counterparts. During the same period, the proportion of independent hospitals decreased. We also found that inpatient prices among nonprofit hospitals were consistently higher and grew faster than for-profit hospitals. Heterogeneity in inpatient price levels and growth between 2012 and 2021 suggest a potential avenue for state and policymakers to take a targeted approach to curtailing hospital prices.

## Data and methods

### Data sources

We used de-identified, commercial claims data from people with employer-sponsored insurance between 2012 and 2021 from the Health Care Cost Institute (HCCI). The HCCI data cover approximately 30% of employer-sponsored insured (ESI) people in the United States—approximately 40 million enrollees per year. Each claim record includes the price that insurers negotiated with the health care provider, as well as information about any diagnoses and procedures associated with the claim, the Diagnosis Related Group, underlying patient demographics, and encrypted hospital identifiers. We supplemented the HCCI data with information on hospital characteristics from the Hospital Cost Report Information System (HCRIS Cost Reports) and the American Hospital Association Annual Surveys.

We constructed a cross-sectional dataset on nonfederal, short-term acute care hospitals, including critical access hospitals. We limited inpatient admissions to individuals aged less than 65 years and excluded inpatient claims with evidence that the health plan was not the primary payer. Using the HCCI data, we aggregated facility claim lines to the admission level and used the sum of the allowed amounts as our measure of price for each inpatient visit. We excluded inpatient admissions if the summed allowed amounts were less than or equal to $1. We excluded government hospitals from our sample. Our primary outcome of interest was the nominal price paid for inpatient hospital care by commercial health plans. We opted to use nominal prices as our primary outcome because hospital prices are negotiated and generally set before the realized inflation rate in the economy. Furthermore, US consumer goods experienced very high inflation rates between 2020 and 2021, which could greatly overstate the role of inflation in hospital inpatient prices.

A challenge in comparing prices across hospitals is variation in inpatients’ allowed amounts driven by differences in the mix of inpatient services provided and differences in negotiated prices. Following previous literature, we addressed this issue by constructing a service mix-adjusted price index for each hospital that controlled for the mix of treatments provided^5,7,8^ (see Appendix A6 for more details). We limited our analytical sample to hospitals with at least 11 inpatient admissions per calendar year. Our secondary outcome of interest was a case mix index (CMI), the average relative weight of a hospital's inpatient discharges in the HCCI dataset, for each hospital-year observation. The CMI provides a descriptive measure of the complexity of each hospital's inpatient admissions per calendar year. Following the methodology developed by the Centers for Medicare and Medicaid Services, the CMI is calculated by summing the Medicare Severity–Diagnosis Related Group weight for each inpatient admission and dividing by the number of inpatient admissions.^9^

### Statistical methods

Our descriptive analyses examined trends in adjusted inpatient price among observable hospital characteristics—that is, health system affiliation and profitability characteristics. Descriptive price analyses were limited to hospitals with at least 11 inpatient admissions per year. All observations were weighted by the number of HCCI admissions per hospital-year. We defined cumulative price growth as the percentage change relative to 2012 baseline hospital prices.

## Results

The share of independent general acute care hospitals has continuously decreased year to year during our study period. Between 2012 and 2021, the share of independent general acute care hospitals decreased from 31.5% of short-term general acute care hospitals in 2012 to 22.3% in 2021. On the other hand, the share of system-affiliated hospitals, particularly among nonprofit hospitals, increased during this time period from 50.6% in 2012 to 62.5% in 2021, corresponding to a 23.4% increase (see Appendix Figure A1).

**Figure 1.**
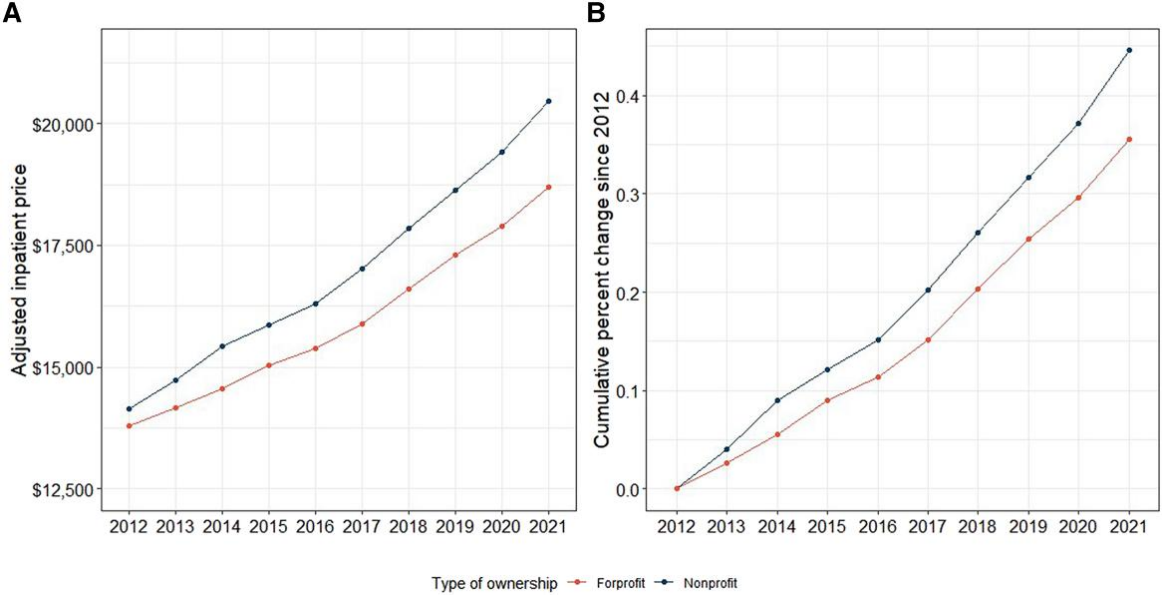
A and B: Price trend and growth between nonprofit vs for-profit hospitals. Sources: Authors’ analysis of data from the Health Care Cost Institute (HCCI), the American Hospital Association, and Centers for Medicare and Medicaid Services. All hospital-year units are weighted based on corresponding number of inpatient admissions in the HCCI data.

In 2012, inpatient hospital prices among nonprofit hospitals were 2.5% higher than for-profit hospitals ($14 152.54 and $13 803.99, respectively) (Figure 1A). Between 2012 and 2021, prices among nonprofit hospitals grew faster than for-profit hospitals. The 10-year rate of inpatient price growth was 45% among nonprofit hospitals compared to a 35% increase among for-profit hospitals. By 2021, nonprofit hospital inpatient prices were 9% higher than for-profit hospitals ($20 457.32 and $18 702.93, respectively) (Figure 1B). We observed a similar trend among hospital characteristics when using 2021 dollars (see Appendix Figure A2).

**Figure 2.**
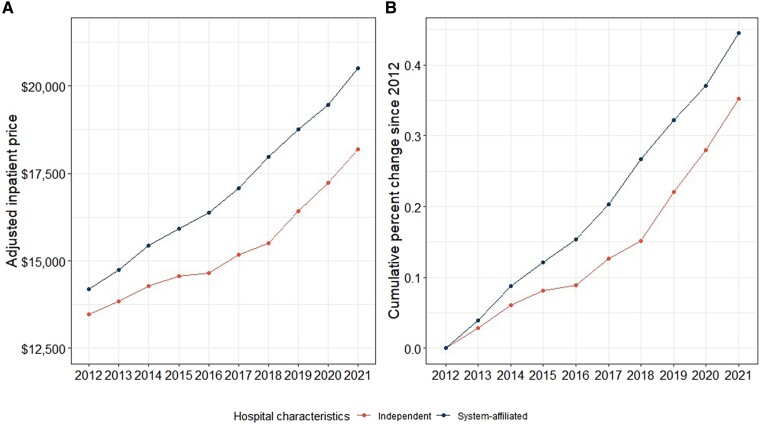
A and B: Price trend and growth between system-affiliation vs independent hospitals. Sources: Authors’ analysis of data from the Health Care Cost Institute (HCCI), the American Hospital Association, and Centers for Medicare and Medicaid Services. All hospital-year units are weighted based on corresponding number of inpatient admissions in the HCCI data.

Inpatient prices among system-affiliated hospitals were associated with higher prices compared with independent hospitals across all years in our study period. In 2012, the average inpatient price among system-affiliated hospitals was $14 194.40 compared to $13 460.50 among independent hospitals. By 2021, inpatient prices among system-affiliated hospitals grew to approximately $20 505.76, nearly 13% higher than their independent counterparts ($18 196.90) (Figure 2A). For most of the 10-year window, the rate of price growth was similar between system-affiliated and independent hospitals (Figure 2B). Faster price growth among system-affiliated hospitals was observed between 2015 and 2018, leading to the greater difference in inpatient prices between the 2 types of hospitals in 2021.

**Figure 3.**
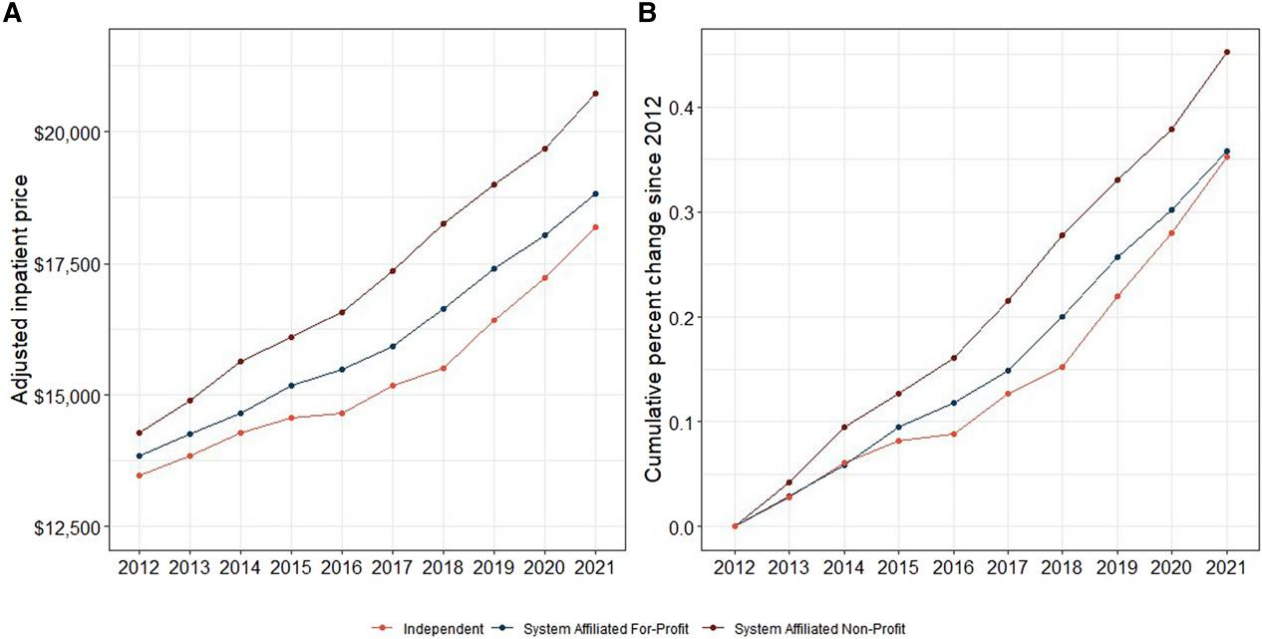
A and B: Price trend and growth between profitability and system affiliation. Sources: Authors’ analysis of data from the Health Care Cost Institute (HCCI), the American Hospital Association, and Centers for Medicare and Medicaid Services. All hospital-year units are weighted based on corresponding number of inpatient admissions in the HCCI data.

Inpatient prices for both system-affiliated nonprofit and system-affiliated for-profit hospitals were higher than for all independent hospitals during the 10-year window (Figure 3A). For example, the average inpatient prices in 2021 for independent, system-affiliated for-profit, and system-affiliated nonprofit hospitals were $18 196.90, $18 811.70, and $20 731.95, respectively. Among system-affiliated nonprofit hospitals, inpatient prices grew faster (45.2%) from 2012 to 2021 than system-affiliated for-profit and independent hospitals (35.8% and 35.2%, respectively) (Figure 3B). Although independent hospitals’ inpatient prices fluctuated during the 10-year period, they remained consistently lower than system-affiliated hospitals, regardless of profit status.

**Figure 4.**
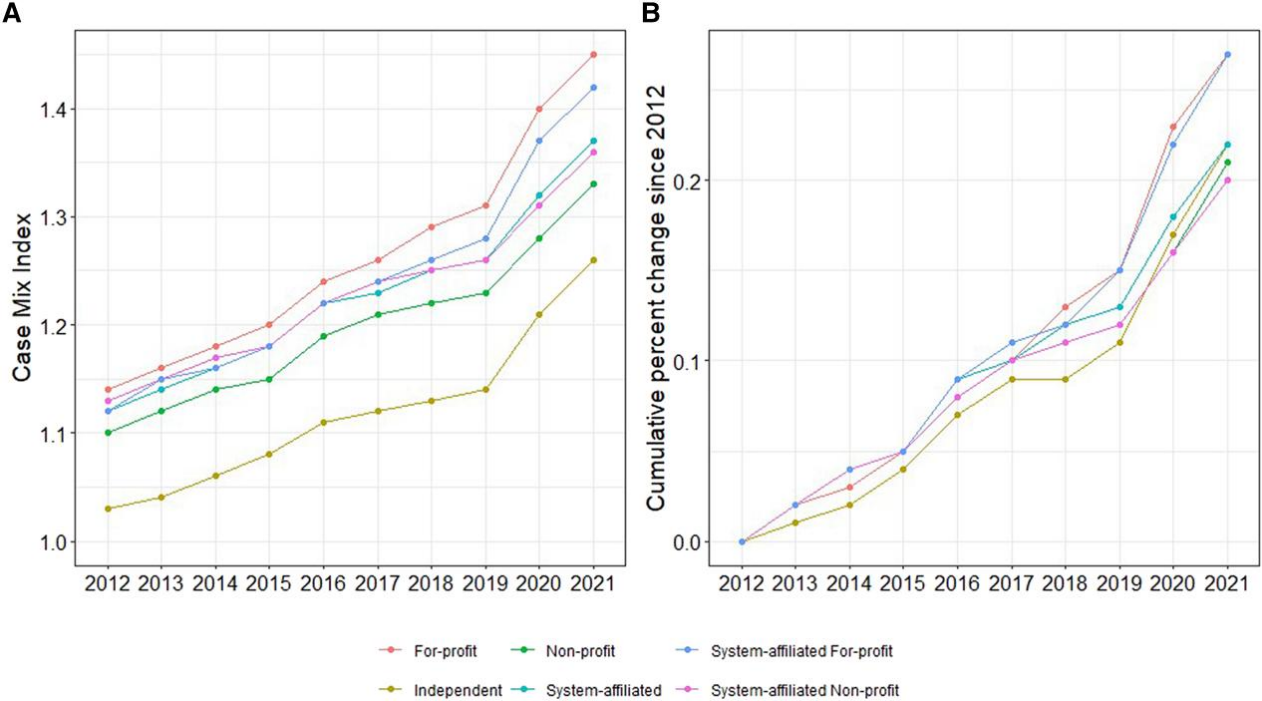
A and B: Case mix trend. Sources: Authors’ analysis of data from the Health Care Cost Institute (HCCI), the American Hospital Association, and Centers for Medicare and Medicaid Services. Hospital categorical values are not mutually exclusive.

Overall, hospitals’ CMI grew between 2012 and 2021 for all hospital characteristics (Figure 4A), suggesting that admissions became more complex over the period for all hospitals. For example, the CMI grew faster in 2020 and 2021 for all hospitals due to the COVID-19 pandemic and correspondingly more complex inpatient admissions. For-profit hospitals had the highest CMI during the study period, while independent hospitals had the lowest CMI during the study period. The CMI growth rate was the fastest among system-affiliated for-profit hospitals from 2012 to 2021 (26.6%) (Figure 4B). The CMI growth rates among other hospital characteristics, such as nonprofit and independent hospitals, were similar (21.2% vs 22.0%, respectively). Despite slightly different CMI levels, the trend in CMI growth rates was comparable across all hospital characteristics between 2012 and 2021. We did not observe diverging differences in CMI among for-profit vs nonprofit or independent vs system-affiliated during our study period (see Appendix Table A3 for percentage differences by group).

## Discussion

Our study found that, during this period, inpatient prices paid by commercial payers have grown faster, particularly among system-affiliated hospitals and particularly nonprofit system-affiliated hospitals. We did not find an association in growth trends between inpatient prices and the complexity of admissions as measured by a CMI, which suggests that case mix is not a driver of differential inpatient price growth during our study period.

Over the study period, the share of independent hospitals decreased between 2012 and 2021, suggesting that the changing composition of short-term general acute care hospitals is associated with a wave of hospital mergers and consolidations. We also observed that the share of system-affiliated and nonprofit hospitals located in highly concentrated hospital referral regions, defined as a Herfindahl-Hirschman index greater than 2500, was elevated compared with other characteristics and grew over time (see Appendix Table A4). Our descriptive results suggest that growth in highly concentrated hospital markets could be a potential driver in hospital inpatient price growth during this time.

Descriptive results from our study should be interpreted as merely associations and do not imply causality between inpatient hospital prices (corresponding growth rates) and observable hospital characteristics. Our study is limited to prices for inpatient admissions and corresponding case mix indices among the ESI population. Future studies should expand to include prices of other lines of services, including those rendered at hospital outpatient departments.

Results from our descriptive study found that not only have inpatient prices among nonprofit hospitals remained higher than for-profit hospitals but they also grew faster between 2012 and 2021. Additionally, nonprofit hospitals do not appear to be treating more complex inpatient admissions than for-profit hospitals in the ESI population. Findings from our study raise questions on the paradox of nonprofit hospitals and inpatient prices. Nonprofit hospitals are exempted from income, property, and sales taxes in exchange for providing community benefits or charity care.^10^ Despite favorable tax status, a body of empirical studies have found inconclusive evidence on whether nonprofit hospitals provide higher levels of community benefits or charity care compared with for-profit or government hospitals. A cross-sectional study on charity care among hospitals in 2018 yielded null effects of charity care provision among nonprofit hospitals compared with for-profit hospitals.^11^ Nevertheless, a recent study found that only 3% of hospital mergers between 2007 and 2020 were challenged by the Federal Trade Commission.^4^ Future research on hospital inpatient price differentials should analyze other hospital characteristics to better understand drivers of higher inpatient prices among nonprofit hospitals.

## Conclusion

State and federal policymakers and advocates have increasingly pointed to market consolidation and oversight of nonprofit hospitals as opportunities to bring about change in hospital spending. The 10-year period from 2012 through 2021 saw an increase in market concentration, and numerous studies have found links between mergers and higher inpatient prices.^5-7^ Despite arguments that mergers improve quality of care through better coordination, research has shown that quality improvements are more common within competitive hospital markets.^12^

With hospitals playing a substantial role in the health care system overall and in health care spending specifically, it is important to better understand the dynamics of hospital prices. Our analyses document variation among inpatient prices and price growth among observable hospital characteristics. That hospital prices and price growth varied by type of hospital suggests that public and private policymakers aiming to rein in health spending need to craft solutions that address this variation. Policies need to be targeted and nuanced to have the greatest impact to improve poorly functioning hospital markets and to avoid unintended consequences.

## Supplementary Material

qxae140_Supplementary_Data

## Data Availability

The raw administrative claims data required to reproduce the above findings cannot be shared, but more information about how to access the Health Care Cost Institute's multi-payer, longitudinal commercial claims dataset is available on HCCI's website.
