# Supplementary material for: Commercial inpatient hospital price growth driven by system affiliation and nonprofit-status hospitals
Source: Health Aff Sch. 2024 Nov 1;2(11):qxae140. doi: 10.1093/haschl/qxae140 (PMC11559425; doi:10.1093/haschl/qxae140)
Supplement: qxae140_Supplementary_Data [file qxae140_supplementary_data.zip › HAScholars_appendix_final.docx]

**Appendices**

**Figure A1: Proportion of short-term general acute care hospitals between 2012 and 2021**


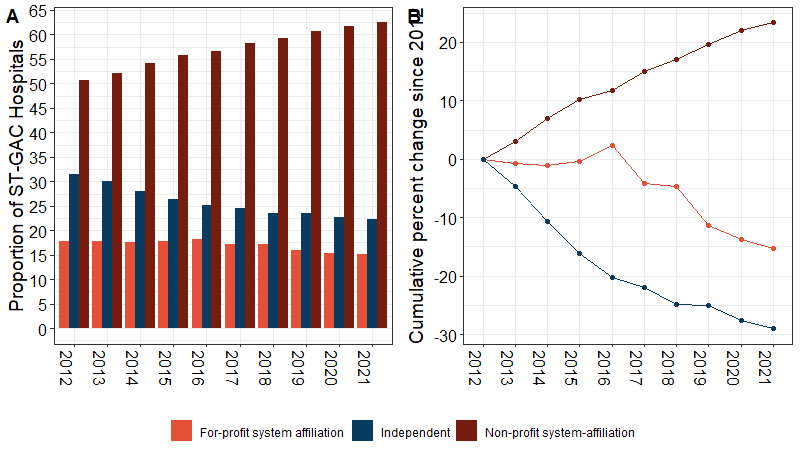


**Sources:** Authors’ analysis of data from the American Hospital Association.

**Figure A2: Hospital inpatient price trend in 2021 dollars**


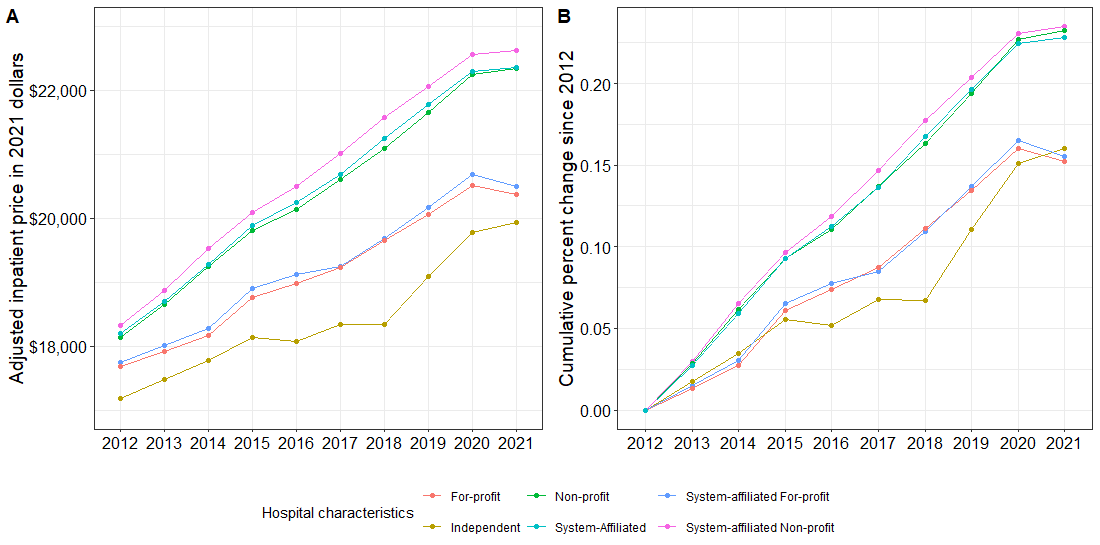


**Table A3: Percent difference in Case Mix Index by hospital characteristics**

| **Year** | **For-profit vs. non-profit** | **System vs. Independent** |
| --- | --- | --- |
| 2012 | 3.8% | 8.3% |
| 2013 | 3.7% | 9.7% |
| 2014 | 3.6% | 9.8% |
| 2015 | 3.6% | 8.9% |
| 2016 | 3.8% | 10.1% |
| 2017 | 4.2% | 10.0% |
| 2018 | 5.1% | 10.7% |
| 2019 | 6.6% | 10.4% |
| 2020 | 9.5% | 9.2% |
| 2021 | 9.2% | 8.7% |

**Note on percent difference interpretation:** In 2012, the average case mix index (CMI) among for-profit hospitals was 3.8% higher than non-profit hospitals. In 2012, the average case mix index (CMI) among system-affiliated hospitals was 8.3% higher than independent hospitals.

**Table A4: Percent of hospitals in highly concentrated market (HHI >= 2500)**

| **Year** | **Independent** | **System-affiliated Non-Profit** | **System-affiliated For-Profit** |
| --- | --- | --- | --- |
| 2012 | 48.88% | 52.36% | 43.66% |
| 2013 | 52.73% | 57.97% | 49.33% |
| 2014 | 53.94% | 60.59% | 53.58% |
| 2015 | 49.52% | 56.40% | 53.05% |
| 2016 | 51.45% | 58.98% | 55.09% |
| 2017 | 52.4% | 59.93% | 55.97% |
| 2018 | 54.36% | 62.95% | 58.91% |
| 2019 | 58.41% | 64.47% | 60.48% |
| 2020 | 54.40% | 64.26% | 58.32% |
| 2021 | 54.72% | 64.46% | 56.67% |
| Growth rate (2012 to 2022) | 11.95% | 23.11% | 29.79% |

**Figure A5: Hospital inpatient price trend for all short-term general acute care hospitals**


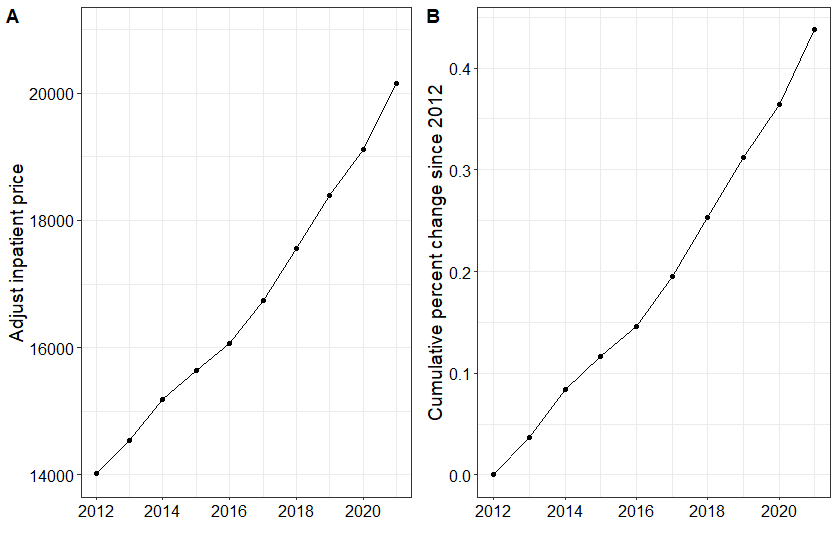


**Appendix A6: Measuring Hospital Prices**

Our primary outcome of interest was inpatient hospital prices. we derived prices from the total allowed amounts reported in the HCCI data. We constructed an inpatient price index for each hospital that controls for the mix of treatments provided. This approach follows work in Gowrisankaren et al. (2015), Cooper et al. (2019); service mix adjustment is particularly in this context because the mix of treatments provided by hospitals can change as competitive dynamics change (e.g., after a closure or after a merger). To minimize the influence of outliers in the analyses, we exclude admissions with a length-of-stay above the 99^th^ percentile within a DRG, or admissions with prices below the 1^st^ or above the 99^th^ percentile.

Our price index measures the amount paid for patient *i* in hospital *h* with DRG *d* in time *t,* where *t* is measured in calendar year intervals*.* We begin by regressing hospital payments (*p_i,h,d,t_*) on hospital-time fixed effects (*α_h,t_*), patient characteristics (*X_i,h,d,t_*) and DRG mix (*γ_d_*). Patient characteristics included age (in 6 age bins) and sex:

*p_i,h,d,t_ = α_h,t_ + β*X_i,h,d,t_ + γ_d_ + ε_i,h,d,t_*

where *ε_i,h,d,t_* is the error term. Next, we captured hospital fixed effects ($\hat{\alpha}$*_h,t_*) and construct a hospital price index given average patient characteristics ($\bar{X}$ and DRG indicators $\bar{d}$):

$\hat{p}$*_h,t_ =* $\hat{\alpha}$*_h,t_ +* $\hat{\beta}$***$\bar{X}$*_i,h,d,t_ +* $\bar{d}\hat{\gamma}$*_d_*
